# Pharmacological Modulation of Radiation Damage. Does It Exist a Chance for Other Substances than Hematopoietic Growth Factors and Cytokines?

**DOI:** 10.3390/ijms18071385

**Published:** 2017-06-28

**Authors:** Michal Hofer, Zuzana Hoferová, Martin Falk

**Affiliations:** Department of Cell Biology and Radiobiology, Institute of Biophysics, v.v.i., Czech Academy of Sciences, Královopolská 135, 61265 Brno, Czech Republic; hoferovaz@centrum.cz (Z.H.); falk@ibp.cz (M.F.)

**Keywords:** acute radiation syndrome, radioprotectors, radiomitigators, hematopoiesis

## Abstract

In recent times, cytokines and hematopoietic growth factors have been at the center of attention for many researchers trying to establish pharmacological therapeutic procedures for the treatment of radiation accident victims. Two granulocyte colony-stimulating factor-based radiation countermeasures have been approved for the treatment of the hematopoietic acute radiation syndrome. However, at the same time, many different substances with varying effects have been tested in animal studies as potential radioprotectors and mitigators of radiation damage. A wide spectrum of these substances has been studied, comprising various immunomodulators, prostaglandins, inhibitors of prostaglandin synthesis, agonists of adenosine cell receptors, herbal extracts, flavonoids, vitamins, and others. These agents are often effective, relatively non-toxic, and cheap. This review summarizes the results of animal experiments, which show the potential for some of these untraditional or new radiation countermeasures to become a part of therapeutic procedures applicable in patients with the acute radiation syndrome. The authors consider β-glucan, 5-AED (5-androstenediol), meloxicam, γ-tocotrienol, genistein, IB-MECA (*N*^6^-(3-iodobezyl)adenosine-5’-*N*-methyluronamide), Ex-RAD (4-carboxystyryl-4-chlorobenzylsulfone), and entolimod the most promising agents, with regards to their contingent use in clinical practice.

## 1. Introduction

Radiation accidents, as well as contingent terrorist attacks using ionizing radiation sources, can result in serious health damage whose manifestations are designated as the acute radiation syndrome (ARS). Depending on the absorbed radiation dose, the manifestation of ARS takes place in different organ systems as individual organ syndromes, namely hematopoietic, gastrointestinal, cutaneous, and neurovascular [1]. Not surprisingly, both the topics of “radioprotectors for use prior to irradiation” and “therapeutic agents for post-exposure treatments” (radiomitigators) enjoy top priority among the research areas for radiological nuclear threat countermeasures [2]. Although endeavors aimed at developing medically effective radiation countermeasures (including both the radioprotectors and radiomitigators) were initiated more than fifty years ago, only two radiation countermeasures, namely Neupogen^®^ and Neulasta^®^, have been recently approved by the United States Food and Drug Administration (FDA) as radiomitigators [3,4].

Hematopoietic growth factors are proteins that regulate growth and differentiation of red and white blood cells. Cytokines are proteins of low molecular weight that exert a stimulating or inhibiting influence on the proliferation, differentiation, and function of cells of the immune system. Both Neupogen^®^ and Neulasta^®^ are granulocyte colony-stimulating factor (G-CSF)-based drugs, made to improve the pharmacokinetic properties of G-CSF [5]. G-CSF belongs to hematopoietic growth factors which, together with cytokines, have been intensively tested and evaluated for modulation of the acute radiation damage e.g., [6,7], and have also been used for the treatment of radiation accident victims [8]. Nevertheless, comparable attention has also been paid by radiation researchers to substances not counted among hematopoietic growth factors or cytokines and/or their derivatives. As shown mostly in animal studies, there exists a wide spectrum of such substances which are often effective in modulating ARS, as well as being relatively non-toxic and cheap. This review summarizes important pieces of information on these agents and emphasizes their potential for incorporation into the treatment schemes of patients with ARS.

## 2. Immunomodulators

Immunotherapy is defined as “treatment of disease by inducing, enhancing, or suppressing an immune response”. A number of immunomodulators inducing and/or enhancing the immune response, which are represented by an array of various preparations, have been tested with the aim of modulating ARS.

### 2.1. β-Glucan

Glucans, especially β-glucan, belong to the most studied immunomodulators both generally, and in the field of the pharmacological modulation of radiation damage. β-glucans are known as cell wall constituents of bacteria [9], yeast [10], fungi [11], and plants [12]. Early hematological studies have revealed that β-glucan stimulates proliferation of non-irradiated mouse pluripotent stem cells, as well as of several hematopoietic progenitor cell lineages, namely those of granulocytes, granulocytes/macrophages, macrophages, and erythrocytes e.g., [13]. Several studies performed, especially by Patchen and McVittie (Bethesda, MD, USA) and Hofer and Pospíšil (Brno, Czech Republic), have shown that the hematopoiesis-stimulating effects of β-glucan can be successfully employed in treating hematopoietic ARS in mice. An important feature of the use of β-glucan in irradiated experimental was the possibility of its profitable administration both prior to and after irradiation, i.e., as radioprotector or radiomitigator [14,15,16,17,18,19,20,21,22,23,24,25]. The use of β-glucan in combined-treatment regimen has also turned out to be successful—mutually potentiating effects of β-glucan have been observed following its combined administration with the chemical radioprotectors cystamine or WR-2721 [26,27]. A three-drug combination treatment of β-glucan, WR-2721, and selenium has shown a positive outcome as well [28]. β-glucan has also been successfully combined with G-CSF [29] or diclofenac, a cyclooxygenase inhibitor [30,31]. Many details concerning the experiments summarized in this paragraph, including information about the enhancement of survival of lethally irradiated experimental animals by β-glucan in some of these studies, can be found in a separate detailed review [32].

Later studies have added new understanding and have confirmed the above-mentioned ability of β-glucan to stimulate hematopoiesis and enhance survival in radiation-exposed animals. In 2006, Cramer et al. [33] revealed the role of complement in mediating the hematopoietic recovery after radiation-induced injury. In 2011, Salama [34] emphasized the possibility to administer, with a positive outcome, β-glucan to irradiated rats orally. In their thorough study from 2013, Pillai and Devi [35] examined the effects of pre-irradiation β-glucan administration, in which cytological and biochemical parameters were included, besides post-irradiation survival and hematopoiesis. Further, apart from their promising findings, they also examined the non-toxicity of β-glucan. In their review from 2009 on the biological activities of β-glucan, Rondanelli et al. [36] stressed the contingent use of β-glucan both as a prophylactic and as a therapy in cases of nuclear emergencies.

### 2.2. 5-Androstenediol (5-AED)

5-androstenediol (5-AED) is a natural adrenocortical steroid hormone. AED has been found to stimulate the innate immune system and, therefore, it is counted among immunomodulators. The first report on the hematopoietic and immune system stimulation observed in γ-irradiated mice is from 2000—both pre- and post-irradiation administration of 5-AED has produced stimulation of myelopoiesis and enhancement of resistance to infection in irradiated animals [37]. In the following study, a stimulation of cells of the immune system, like monocytes, natural killer cells, eosinophils, and basophils, has been observed following administration of 5-AED [38]. Further experiments have revealed that 5-AED is effective at both subcutaneous and oral administration routes, and that the radioprotective efficacy of the drug is accompanied by low toxicity [39]. As one of the mechanisms of the hematopoiesis-stimulating effects of 5-AED, an induction of amplified production of G-CSF (when 5-AED was administered either as a radioprotector or as a radiomitigator) has been reported [40]. Comparative experiments have shown that the radioprotective efficacy of 5-AED is unique among ten selected different steroids [41]. Subsequently, studies on mice have been supplemented by experiments on non-human primates—a multilineage stimulation of hematopoiesis in irradiated rhesus monkeys has been found [42]. This knowledge has been obtained even when the irradiated monkeys were otherwise clinically unsupported [43]. An induction of nuclear factor-κB activation has been found as the mechanism of enhanced survival of irradiated human hematopoietic progenitors in the presence of 5-AED [44]. To further elucidate the mechanisms of hematopoiesis-stimulating effects of 5-AED, expression of a number of hematopoietic growth factors and cytokines in 5-AED-treated mice has been evaluated. An increased expression following the injection of 5-AED has been referred to as granulocyte-macrophage colony-stimulating factor (GM-CSF), interleukin-2 (IL-2), interleukin-3 (IL-3), interleukin-6 (IL-6), and interleukin-10 (IL-10) in the spleen, as well as to G-CSF, GM-CSF, interferon-γ (IFN-γ), thrombopoietin (TPO), IL-2, IL-3, IL-6, IL-10, and interleukin-12 (IL-12) in the bone marrow [45]. The significant role of G-CSF in mediating the effects of 5-AED has been confirmed by a study in which G-CSF antibody was used for abrogating the radioprotective efficacy of 5-AED [46]. A synergistic action of 5-AED and TPO has been shown in mice suffering from hematopoietic ARS [47]—in this study, a 20.1-fold increase in the life-saving short-term repopulating cells in the bone marrow has been observed in the 5-AED + TPO-treated mice [47]. A sequential injection of 5-AED, comprising one pre-irradiation administration and twice weekly injections for three weeks post-irradiation, have been reported to be very successful in treating the radiation-induced myelosuppression [48]. The authors have stated that “5-AED can be a significant therapeutic candidate for the management of ARS, particularly in a mass casualty scenario where rapid and economic intervention is important [47]”. 5-AED is now in advanced development and has been granted FDA investigational new drug (FDA IND) status [49]. First positive clinical observations on the safety, tolerability, and hematologic activity of 5-AED in healthy adults have been already reported as well [50].

### 2.3. Other Immunomodulators

This paragraph summarizes data on another immunomodulators tested from the point of view of their abilities to modulate ARS.

Perhaps the oldest immunomodulator studied as a radioprotector is endotoxin, a bacterial lipopolysaccharide. As early as 1958, a report on decreased X-ray mortality in endotoxin-treated mice was published [51]. As an important manifestation of its radioprotective action, an increased number of spleen colony-forming units (used for expression of numbers of hematopoietic stem cells) in endotoxin-pre-treated irradiated mice has been reported [52]. A large amount of literature has been published on the endotoxin’s radioprotective properties. However, because of its severe side effects [53], endotoxin has been gradually abandoned as a potentially usable drug in irradiated humans. On the contrary, reduction of endotoxemia has been considered a desirable effect of drugs used for the treatment of ARS, e.g., with antibiotics [54].

Broncho-Vaxom^®^ is a bacterial lysate. It has been shown to significantly enhance post-irradiation survival in several mouse strains [55]. Subsequent studies have revealed positive hematological effects of Broncho-Vaxom^®^ in sublethally irradiated mice [56,57,58]. Broncho-Vaxom^®^ has been also tested with success in its combined administration with the chemical radioprotector WR-2721 (amifostine) [59,60]. Broncho-Vaxom^®^ has been found to act radioprotectively when administered pre-irradiation [55,56,57,58,59,60], as well as a radiomitigator following its post-irradiation administration [60]. In a later study, Broncho-Vaxom^®^ has been administered to rats in repeated injections comprising one pre-irradiation dose and repeated post-irradiation doses in the course of a three-week period of fractionated irradiation. The drug has been reported to enhance the antioxidant system and to increase the serum γ-globulin content [61].

Trehalose dimycolate is a glycolipid molecule found in the cell wall of *Mycobacterium tuberculosis*. It has been reported to enhance resistance to infection in irradiated neutropenic mice [62]. A synthetic derivative of trehalose dimycolate (trehalose dicornomycolate) has been tested with success in mice with sepsis following irradiation and trauma [63].

An interesting radioprotective combination is that of the oligoelements selenium, zinc, and manganese administered concomitantly with *Lachesis muta* venom. This combination is called “immunomodulator” by the authors and has been tested in both mice [64] and rats [65].

Peptidoglycan is a bacteria cell wall polymer consisting of sugars and amino acids. In a recent study, peptidoglycan was observed to promote survival, as well as to ameliorate intestinal and bone marrow damage in irradiated mice when injected after irradiation [66]. These parameters have been found to be synergistically promoted when the mice were given the chemical radioprotectant WR-2721 pre-irradiation and peptidoglycan post-irradiation [66]. Apart from radioprotection of the hematopoietic and gastrointestinal tissues, a complete prevention of permanent submandibular gland radiation-induced alterations has been reported, following the administration of this radiomodifying mixture of compounds [66].

Other recent studies have been concerned with *Sipunculus nudus* (a species of unsegmented marine worms) polysaccharide. *Sipunculus nudus* polysaccharide, consisting of mannose, rhamnose, galacturonic acid, glucose, arabinose, and fucose, administered before irradiation, has been found to significantly increase survival of irradiated mice [67]. When the substance was tested in Beagle dogs, *Sipunculus nudus* polysaccharide-treated animals have shown, among others, an improved blood picture and an improved hematopoietic activity in the bone marrow [68]. Synergistic effects have been reported for the radioprotective combination of the *Sipunculus nudus* polysaccharide, WR-2721, recombinant human interleukin-11 (rhIL-11), and recombinant human G-CSF (rhG-CSF) in radiation-injured mice [69]. Marked antioxidant effects of *Sipunculus nudus* polysaccharide [67], and its efficacy following its oral administration [68], have been emphasized.

## 3. Prostaglandins and Inhibitors of Prostaglandin Production

Quite surprisingly, both prostaglandins, especially prostaglandin E_2_ (PGE_2_), and inhibitors of prostaglandin production (cyclooxygenase (COX) inhibitors), have been successfully tested regarding their abilities to support recovery of experimental animals from ARS. Therefore, both groups of substances are dealt with in the same section.

Several studies from the 1980s showed radioprotective effects of prostaglandins, particularly PGE_2_ and a synthetic derivative of prostaglandin E_1_, misoprostol, on irradiated gastrointestinal tracts [70,71,72]. These effects might be ascribed to the subsequently confirmed protective action of prostaglandins on the gastrointestinal tissues [73,74]. However, at the approximately same time, articles showing that PGE_2_ stimulates and/or protects erythroid and multilineage hematopoietic progenitor cells [75,76,77] also appeared. Nevertheless, findings on inhibition of myelopoiesis in vivo by PGE_2_ were also published at that time [78,79].

The results mentioned [78,79] help to justify the findings obtained when the action of inhibitors of prostaglandin production (COX inhibitors, non-steroidal anti-inflammatory drugs) in irradiated experimental animals was evaluated. In earlier studies, the radiomodifying effects of non-selective COX inhibitors, inhibiting the synthesis of both cyclooxygenase-1 (COX-1), expressed constitutively in a variety of tissues including the gastrointestinal tract, and cyclooxygenase-2 (COX-2), was tested. This is inducible and responsible for the production of prostaglandins during inflammatory states [80]. In sublethally irradiated experimental animals, hematopoiesis-stimulating effects of non-selective COX inhibitors have been observed when they were administered pre- or post-irradiation, or in the course of the fractionated radiation regimen [81,82,83,84,85,86,87,88,89,90]. However, administration of non-selective COX inhibitors has also been connected to the occurrence of a rather high incidence and intensity of undesirable side effects, especially on the gastrointestinal tissues [90], and a reduced survival of lethally irradiated animals [91,92]. Numerous details on the effects of non-selective COX inhibitors in irradiated animals can be found in an earlier detailed review [93].

Later investigations on the radiomodifying action of COX inhibitors have used a representative of selective COX-2 inhibitors, meloxicam, whose administration preserves the activity of COX-1 and maintains the protective action of prostaglandins in the gastrointestinal tissues [73,74]. Meloxicam has been shown not only to support hematopoiesis in irradiated mice [94,95], but also to enhance the post-irradiation survival of the animals, namely when administered in a mere single dose 1 h after a lethal irradiation [96]. Thus, favorableness of the use of selective COX-2 inhibitors as radiomitigators has been confirmed.

In a recent study, Hoggatt re-opened investigations on hematological and radiomodifying effects of pharmacological interventions into the metabolism of PGE_2_. They stated that PGE_2_ enhances hematopoietic stem cell homing, survival, and proliferation [97]. Taking into account all of the available knowledge on the modulation of PGE_2_ signaling post-irradiation, as well as their own experimental results, Hoggatt et al. found that an increased survival and stimulation of hematopoiesis in irradiated mice can be obtained both by an administration of PGE_2_, and following the treatment with the selective COX-2 inhibitor meloxicam, but that the effectiveness of the therapies depends on the timing of the injections [98].

## 4. Herbal Extracts

Herbal extracts tested for radioprotective and radiomitigating properties comprise preparations from a number of plants. Their action is complex and comprises their antiemetic activity, anti-inflammatory activity, antimicrobial activity, antioxidant activity, hematopoietic stimulation, immunostimulant activity, metal chelation activity, and wound healing activity [99]. In a thorough review from 2005, radioprotective/radiomodifying effects of the extracts from the following plants are summarized with a number of citations: *Acanthopanax senticosus*, *Aegle marmelos*, *Ageratum conyzoides*, *Allium cepa*, *Allium sativum*, *Aloe arborescens*, *Amaranthus paniculatus*, *Angelica sinensis*, *Archangelica officinalis*, *Centella asiatica*, *Curcuma longa*, *Gingko biloba*, *Glycyrhizza glabra*, *Hipophae rhamnoides*, *Hypericum perforatum*, *Lycium chinense*, *Mentha arvensis*, *Mentha piperita*, *Moringa oleifera*, *Ocimum sanctum*, *Panax ginseng*, *Podophyllum hexandrum*, and *Tinospora cordifolia* [99]. Generally, herbal extracts are considered successful in treating symptoms of ARS, and their research has continued until the present time. e.g., an extract from *Cordyceps sinensis* has been observed to protect against both the bone marrow and intestinal radiation-induced injuries [100]. Pre-irradiation administration of an extract from *Podophyllum hexandrum* has shown radioprotective efficacy, which has been further enhanced by post-irradiation application of an extract from *Picrorhizza kurroa* [101]. A semi-purified fraction of *Podophyllum hexandrum*, REC-2001, has been shown to significantly enhance survival of lethally irradiated mice [102]. Stimulatory effects in both the immune tissues in irradiated mice have been reported following the administration of the extract from *Vernonia cinerea* [103]. Recently, a potent radioprotective effect of a herbal drug prepared from *Rosa canina*, *Urtica dioica*, and *Tanacetum vulgare*, has been observed [104].

## 5. Amifostine

Amifostine (WR-2721) will be dealt with here briefly. Amifostine has been the most exhaustively studied classical chemical radioprotector from the point of view of its ability to protect against ARS because of its high radioprotective efficacy, as summarized in many reviews e.g., [105,106,107]. For interested readers, we refer them to a rich literature on amifostine and other chemical radioprotectors. Despite the comprehensive number of studies, amifostine has not been approved for the treatment of ARS in humans because of its undesirable side effects, and has found its use in radiation oncology [108,109]. These issues are, however, outside the topic of this review. Nevertheless, attention is still paid to amifostine from the point of view of its contingent use in treating of ARS—successful attempts have been made recently when administering mice with low doses of amifostine (30 or 50 µg/kg) plus γ-tocotrienol in a combined prophylactic modality [110]. Combined approaches using low amifostine doses might enable the use of this radioprotector in humans in the treatment of ARS.

## 6. Antioxidants

A rather wide spectrum of substances, mostly naturally occurring ones, will be addressed in this section Their common feature their ability to protect against or to treat radiation damage by scavenging radiation-induced free radicals. These natural antioxidants are generally less effective radioprotectors in comparison with amifostine and other classical chemical radioprotectors, but may provide a longer window of protection [111] and are often non-toxic.

### 6.1. Vitamin E Family Members

Vitamin E represents a family of compounds that is divided into two subgroups called tocopherols and tocotrienols; both function as important antioxidants [112]. They differ chemically in that tocotrienols contain three double bonds in their isoprenoid side chain, while tocopherols do not [113]. Tocotrienols have superior antioxidant activity compared with tocopherols [114]. There exist ample literature on the radioprotective and radiomitigating action of vitamin E family members, whose detailed summarization exceeds the extent of this review. Therefore, for each subgroup, selected examples of experimental findings will be shown and, finally, further research literature will be recommended.

Concerning tocopherols, three compounds have been tested for their contingent modulating effects on ARS. α-tocopherol has been found to enhance both survival and ARS symptoms when administered both pre- and post-irradiation [115,116,117]. As the mechanism of the α-tocopherol’s radiomitigative effect, an enhancement of cell-mediated immunity has been proposed [118]. α-tocopherol-mono-glucoside is a water-soluble glycosylated derivative of α-tocopherol. When administered post-irradiation, α-tocopherol-mono-glucoside has been demonstrated to increase survival [119] and to stimulate hematopoiesis in mice [120,121]. Attention has also been paid to α-tocopherol succinate, the hemisuccinate ester derivative of α-tocopherol. α-tocopherol succinate has been observed to enhance survival of irradiated mice, including mice irradiated with doses causing the gastrointestinal ARS [122,123]. As for hematopoiesis-modulating action of α-tocopherol succinate, it has been reported that its radioprotective efficacy is mediated through the induction of G-CSF production [124].

Regarding tocotrienols, two vitamin E family members have been investigated from the point of view of their abilities to influence ARS, namely δ-tocotrienol and γ-tocotrienol. δ-tocotrienol possess very high antioxidant activity [125], and has been also shown to protect hematopoiesis and increase survival of irradiated mice [126,127]. In recent years, attention has been paid especially to γ-tocotrienol, another vitamin E derivative with a high antioxidant ability [128]. From the perspective of its contingent use in treating ARS, the hematopoiesis-stimulating [129] and survival-enhancing efficacy of γ-tocotrienol [130] should be emphasized. An important role of G-CSF in mediating the radioprotective effects of γ-tocotrienol has been shown when using G-CSF antibodies [131]. Recently, the radioprotective efficacy of γ-tocotrienol has also been confirmed in non-human primates [132].

Many more details and literature on the radiobiological properties of tocopherols and tocotrienols can be found in a 2013 review [133]. A recent separate detailed review has been devoted to γ-tocotrienol [134]. It has been stated that a single administration of γ-tocotrienol without any supportive care was equivalent, in terms of improving hematopoietic recovery, to multiple doses of Neupogen and Neulasta (both G-CSF-based drugs) with full supportive care (including blood products) in the non-human primate model [summarized in 134]. γ-tocotrienol has been categorized by Singh et al. among “promising molecules at advanced stages of development” [49].

### 6.2. Selenium-Containing Compounds

Several selenium derivatives have been investigated for their radioprotective effects; perhaps the most intensively studied selenium compounds have been sodium selenite and selenomethionine. Selenomethionine, which is a naturally occurring derivative of low toxicity, can be found in soy, grains, and legumes [135]. Expressive and significant survival benefits in irradiated mice have been obtained when either sodium selenite or selenomethionine were administered to mice 24 h or one hour before, or 15 min after, irradiation [136]. Concerning the mechanisms of the radiomitigating effects of the post-irradiation administration of selenium, an enhancement of functions of immunocompetent cells has been proposed [137]. There exists a great amount of research on the ability of selenium compounds to modulate radiation damage where many details can be found [138,139]. Recent findings showing that selenium protects intestinal tissue against ionizing radiation [140] suggest the usefulness of selenium administration in the intestinal ARS.

### 6.3. Other Antioxidative Compounds

Many antioxidative compounds have been tested for their radiomodifying properties. This paragraph gives examples of some of them. Their common characteristics are mostly very low toxicity, as well as the possibility of their peroral administration.

Vitamin C (ascorbic acid) has been reported to improve the bone marrow status and survival of irradiated mice [141]. Further, mice given peroral vitamin A or its precursor, β-carotene, have shown reduced morbidity and mortality [142]. Recently, protective effects of α-lipoic acid on radiation-induced small intestine injury has been found in mice [143], suggesting the potential use of this antioxidative compound in the treatment of the intestinal ARS.

An interesting contribution to the topic of the combined pharmacological approach to the modulation of radiation damage has been provided by Wambi et al. who tested the efficacy of a dietary supplement consisting of L-selenomethionine, vitamin C, vitamin E succinate, α-lipoic acid, and *N*-acetyl-cysteine. This supplement positively influenced hematopoiesis and survival of irradiated mice when given either before or after their irradiation with X-rays [144], or when administered after exposure of the animals to proton irradiation [145].

Antioxidant nutrients are considered in more detail in a review [146].

## 7. Other Compounds Tested as Radioprotectors or Radiomitigators

### 7.1. Genistein

Genistein is a soy isoflavone. Its effects in an irradiated organism are probably complex, including antioxidative [147] and hematopoiesis-stimulating actions [148].

In a report from 2003, genistein has been shown to significantly increase survival of mice when administered in a single preirradiation subcutaneous injection [149]. A complex enhancement of a wide spectrum of bone marrow and blood parameters has been reported when genistein was administered once daily for seven consecutive days before a whole-body irradiation [148]. Hematopoiesis-stimulating effects of genistein have also been confirmed following its single pre-irradiation dose [150]. Details on the radiomodifying actions of genistein are summarized in a separate review [151].

Genistein, administered pre-irradiation has also been successfully combined with captopril, an angiotensin converting enzyme, given to mice in their drinking water on days 1 to 30 after irradiation, as shown by stimulated hematopoiesis and increased survival of mice. For example, whereas the whole-body dose of 8.25 Gy resulted in 0% survival in untreated mice, administration of genistein, captopril, or genistein + captopril increased survival to 72%, 55%, and 95%, respectively [152]. To overcome genistein’s low water solubility, a nanoparticle suspension of genistein has been formulated. This form of genistein has also shown protection of hematopoietic system in irradiated mice [153]. Genistein has been granted FDA IND status [49].

### 7.2. Adenosine Receptor Agonists

The combination of dipyridamole (DP), a drug inhibiting the cellular uptake of adenosine [154], and adenosine monophosphate (AMP), an adenosine prodrug [155], has been used in an attempt to enhance the receptor action of adenosine in a series of studies on irradiated mice. The results of these studies have clearly shown that the pre-irradiation combination of DP and AMP stimulates hematopoiesis and increases survival under conditions of single [156,157,158,159], as well as fractionated irradiation [159,160].

Adenosine receptors, activated non-selectively in the experiments mentioned [155,156,157,158,159,160], exist in four subtypes. Further experimentation, using agonists selected for the individual subtypes, has been aimed at finding out whether stimulation of one of the four subtypes is responsible for the previously observed hematopoiesis-stimulating and radioprotective effects. These experiments are described in detail in a separate review [161]. It follows from the findings that IB-MECA (*N*^6^-(3-iodobenzyl)adenosine-5′-*N*-methyluronamide) stimulates the cycling of hematopoietic progenitor cells [162]. Subsequently, IB-MECA has been shown to support hematopoiesis, as well as the hematopoiesis-stimulating effects of G-CSF in sublethally irradiated mice [163]. Mutually potentiating effects on hematologic parametrs in irradiated mice have been also observed following a concommitant administration of IB-MECA and meloxicam, a cyclooxygenase-2 inhibitor, in a post-irradiation treatment regimen [164]. This series of studies has been completed with the finding that IB-MECA and meloxicam, given in the same therapeutical treatment regimen to lethally irradiated mice, enhance the survival of the exposed animals, each alone or in a combination [165].

### 7.3. More Selected Compounds

Ex-RAD (ON 01210.Na, 4-carboxystyryl-4-chlorobenzylsulfone), is a rather new chemical entity, reported to possess significant radioprotective effects in terms of survival following either subcutaneous [166] or peroral administration routes [167], as well as of ameliorating hematopoietic and gastrointestinal damage [168]. The mechanisms of the radioprotective effects of Ex-RAD involve prevention of p53-dependent and independent radiation-induced apoptosis [162], as well as attenuation of the DNA damage response [169] and the up-regulation of PI3-Kinase/AKT pathways in cells exposed to radiation [170]. Ex-RAD possesses the FDA IND status [49].

Metformin is a biguanide drug used in the treatment of type II diabetes. Recently metformin’s radiomitigating effects when administered 24 h after irradiation alone or in pharmacological combinations [171]. Metformin is approved by FDA for human use and has a well characterized human safety profile [171].

Toll-like receptor 5 (CBLB502, entolimod) is a polypeptide drug derived from salmonella flagellin [172]. It has been shown to enhance survival and protect against hematopoietic and gastrointestinal ARS when administered either before or after irradiation [172,173]. Entolimod has been found to increase the clonogenic potential of the bone marrow cells and to reduce apoptosis in the intestinal crypts [173]. Entolimod has found its use also in mitigation of radiation-induced epithelial damage in a mouse model of fractionated head and neck irradiation [174]. It has been also demonstrated that G-CSF and IL-6 may serve as efficacy biomarkers for this agent [175]. This is an important observation since such biomarkers may be helpful for dose conversion from animal to human, especially in view of the fact that entolimod also possesses the FDA IND status [49].

FGF-2 peptide, a peptide derived from the binding domain of fibroblast growth factor, has been reported to rescue a significant fraction of four strains of mice with the gastrointestinal ARS. Use of FGF-2 peptide has improved crypt survival and repopulation, partially preserved or restored GI function, and has reduced radiation-induced increased plasma endotoxin and pro-inflammatory cytokine levels [176].

Octadecenyl thiophosphate, a small molecule mimic of the growth factor-like mediator lysophosphatidic acid, has been shown to either protect from or mitigate both the hematopoietic and gastrointestinal ARS [177]. Besides direct effects on the gastrointestinal and hematopoietic tissues, octadecenyl thiophosphate has also been found to reduce endotoxin seepage into blood [177].

The gastrointestinal ARS has also been the target of the radioprotective and radiomitigating pharmacological approach using inhibitors of prolyl hydroxylase domain-containing enzymes (PHDs), whose administration has resulted in stabilization of hypoxia-inducible factors (HIFs) protecting important cellular compartments from radiation-induced damage [178]. The role of the PHD/HIF axis in the radiation-induced gastrointestinal toxicity has been recently reviewed and the procedures using PHDs resulting in stabilization of HIFs have been proposed as new class of radioprotectors [179].

## 8. Remarks to Cutaneous Syndrome of Acute Radiation Syndrome (ARS)

Although the cutaneous syndrome of ARS is clinically relevant both for both the radiation victims and radiotherapy-exposed oncological patients, it will be briefly considered separately. In patients with the cutaneous syndrome, pharmacological therapy applying topical or systemic steroids [180,181], or combined pentoxifylline and vitamin E [182] (for the treatment of late consequences of the radiation damage to the skin) has been used. However, current approaches for the treatment of the cutaneous syndrome consist of, among others, non-pharmacological methods like local injections of bone marrow mesenchymal stem cells [183,184] or adipose tissue-derived stromal/stem cells [185]. A more detailed consideration of therapeutical approaches aimed at treatment of the cutaneous syndrome lies outside of the scope of this article.

Agents mentioned in this review which have been used in attempts to modify the course of the acute radiation syndrome (ARS) (not including hematopoietic growth factors and cytokines) are summarized in Table 1.

## 9. Discussion and Conclusions

Though rather rich in content, this review cannot address all publications dealing with the topic of non-hematopoietic growth factor- and non-cytokine-based treatment of ARS. However, it hopefully gives a suitable overview of important pharmacological approaches aimed and protecting from, or mitigating the consequences of, acute radiation doses inducing ARS.

It can be inferred from the literature summarized here that the topic of pharmacological modulation of radiation damage not only has a long history, but is still alive and active. Many compounds seem to be promising from the point of view of their contingent future incorporation into medical procedures aimed at mitigating or protecting from ARS. In the authors’ opinion, β-glucan, 5-AED, meloxicam, γ-tocotrienol, genistein, IB-MECA, Ex-RAD, and entolimod can find their place among the most promising agents, apart from other hopeful compounds recently or currently tested.

An important aspect in considering the contingent use of the individual compounds in human clinical practice is the fact that many of them are non-toxic (or possess only low toxicity), that they are available (or can be made available readily), and that they are often cheap to prepare in even large quantities.

Therefore, the answer to the question from the title of this review, namely is there is a chance for other compounds than hematopoietic growth factors or cytokines in the pharmacological modulation of radiation damage, is “yes, there is a good chance”.

## Figures and Tables

**Table 1:** Summary of agents (not including hematopoietic growth factors and cytokines) used in attempts to modify the course of the acute radiation syndrome (ARS).

Agent or Group of Agents	Predominant Radiomodifying Effect(s)	Reference Number(s)
4-carboxystyryl-4-chlorobenzylsulfone (Ex-RAD)	Prevention of apoptosis	[49,166,167,168,169,170]
5-androstenediol (5-AED)	Immunomodulator, stimulator of hematopoiesis	[37,38,39,40,41,42,43,44,45,46,47,48,49,50]
Adenosine monophosphate (AMP)	Stimulator of proliferation of hematopoietic progenitor cells	[155,156,157,158,159,160]
α-Lipoic acid	Antioxidant	[143,145]
Amifostine (WR-2721)	Free radical scavenger	[59,60,66,69,105,106,107,108,109,110]
β-Glucan	Immunomodulator, stimulator of hematopoiesis	[9,10,11,12,13,14,15,16,17,18,19,20,21,22,23,24,25,26,27,28,29,30,31,32,33,34,35,36]
Broncho-Vaxom	Immunomodulator, stimulator of hematopoiesis	[55,56,57,58,59,60,61]
Captopril	Vasodilator	[152]
Dipyridamole	Enhances adenosine receptor action, stimulator of proliferation of hematopoietic progenitor cells	[154,156,157,158,159,160]
Endotoxin	Immunomodulator, stimulator of hematopoiesis	[51,52,53,54]
FGF-2 peptide	Improvement of regeneration of radiation-induced gastrointestinal damage, reduction of endotoxemia	[176]
Genistein	Antioxidant	[49,147,148,149,150,151,152,153]
Inhibitors of prolyl hydroxylase domain-containing enzymes	Antioxidants	[178,179]
*Lachesis muta* venom	Immunomodulator	[64,65]
Manganese-containing compounds	Antioxidants	[64,65]
Meloxicam, selective cyclooxygenase-2 inhibitor	Inhibitor of prostaglandin production, stimulator of myelopoiesis	[94,95,96,97,98]
Metformin	Antioxidant, modulator of cell renewal	[171]
*N*^6^-(3-iodobenzyl)adenosine-5′-*N*-methyuronamide (IB-MECA)	Stimulator of hematopoietic cell proliferation through adenosine receptor action	[161,162,163,164,165]
*N*-acetyl-cysteine	Antioxidant	[144,145]
Non-selective cyclooxygenase inhibitors (non-selective non-steroidal anti-inflammatory drugs)	Inhibitors of prostaglandin production, stimulators of myelopoiesis	[81,82,83,84,85,86,87,88,89,90,91,92,93]
Octadecenyl thiophosphate	Stimulation of hematopoiesis, reduction of endotoxemia	[177]
Pentoxifylline	Improvement of blood flow properties	[182]
Peptidoglycan	Immunomodulator, ameliorates bone marrow and intestinal radiation-induced damage	[66]
Prostaglandins E and their derivatives	Hematopoietic modulators, radioprotectants of intestinal tissues	[70,71,72,73,74,75,76,77,78,79]
Selenium-containing compounds	Antioxidants	[64,65,135,136,137,138,139,140]
Sipunculus nudus polysaccharide	Immunomodulator, antioxidant	[67,68,69]
Steroids	Antiinflammatory action	[180,181]
Toll-like receptor 5 (CBLB502, entolimod)	Stimulation of proliferation of hematopoietic cells, prevention of apoptosis	[49,172,173,174,175]
Trehalose dimycolate and its derivatives	Immunomodulators	[62,63]
Vitamin C (ascorbic acid)	Antioxidant	[141]
Vitamin A and its precursor	Antioxidant	[142]
Vitamin E and its derivatives	Antioxidants	[49,112,113,114,115,116,117,118,119,120,121,122,123,124,125,126,127,128,129,130,131,132,133,134]
Zinc-containing compounds	Antioxidants	[64,65]
